# Disparities in Female Oncofertility Care in the United States: More Questions Than Answers

**DOI:** 10.3390/life13071547

**Published:** 2023-07-12

**Authors:** Kati A. Turner, Emily E. Spurlin, Patricia T. Jimenez

**Affiliations:** Department of Obstetrics and Gynecology, Washington University, St. Louis, MO 63110, USAspurlin@wustl.edu (E.E.S.)

**Keywords:** fertility preservation, oncofertility, health disparities

## Abstract

As cancer therapies continue to improve, the survival rates of adolescent and young adult patients have increased. Consequently, considering patient quality of life after cancer, including family building, has become an essential aspect of establishing a treatment plan. However, the gonadotoxic nature of many chemotherapeutic agents limits the option of using one’s own gamete for family building. In recent years, significant advancements have been made in oncofertility, particularly vitrification of oocytes. Unfortunately, as with many areas of medicine, health disparities limit those that can access and utilize fertility preservation prior to cancer treatment. This review aims to shed light on existing disparities in oncofertility for female patients, to offer recommendations to enhance education, access, and advocacy, as well as identify potential areas for future research.

## 1. Introduction

With remarkable advances in anti-cancer therapies, adolescent and young adult patients with cancer are surviving into adulthood at increasing rates. As a result, issues related to survivorship, such as family building after completing treatment, are becoming more important. The field of oncofertility has made significant progress in ensuring a reproductive future for these patients, and now it is the standard of care to offer fertility preservation counseling prior to gonadotoxic therapy in patients of reproductive age. Fertility preservation procedures most commonly include sperm, oocyte, and embryo cryopreservation [1]. Additional options for female patients include gonadotropin-releasing hormone agonists (GnRHa) to decrease the risk of chemotherapy-induced ovarian insufficiency when proven methods are not feasible, ovarian transposition to protect the ovaries from the irradiated field when pelvic radiation is utilized, and ovarian tissue cryopreservation, an experimental method of fertility preservation with encouraging results and significant potential, especially for pre-pubertal patients with malignancy [1,2,3].

Several professional societies including the American Society for Clinical Oncology (ASCO), American Society for Reproductive Medicine (ASRM), and American College of Obstetricians and Gynecologists (ACOG), recommend that healthcare providers discuss the possibility of infertility related to cancer therapies and fertility preservation options with patients. Referral to a reproductive specialist should occur as soon as possible before cancer treatment is initiated [1,4,5]. Unfortunately, the literature suggests these recommendations may not have been fully adopted, as the proportion of providers reporting routine discussion of the impact of treatment on fertility and referral of patients to reproductive endocrinology for consultation ranges from 47 to 95%. Several retrospective surveys of patients diagnosed with cancer in adolescence and young adulthood reveal that 56 to 72% recall a discussion regarding the impact of treatment on fertility or fertility preservation prior to beginning treatment [6,7,8]. While the retrospective nature of these studies may raise concerns of recall bias, comparisons of chart-documented discussions and patient recall of conversations regarding infertility related to cancer care or fertility preservation reveal significant discrepancies. Only 52% of patients recalled a discussion when one was documented, and 59% of patients recalled a discussion when none was documented, suggesting that recall bias may artificially diminish or inflate these statistics in roughly equal measure [9].

It is well-established that, outside of an oncofertility context, racial disparities exist in access to and utilization of fertility services. Compared to white women, African American and Hispanic women attempt to conceive for a longer period before seeing a reproductive specialist and face greater difficulty in obtaining an appointment, taking time off from work, and paying for treatment [10]. Black women are most likely to suffer from infertility, yet least likely to seek fertility treatment [11,12,13]. These disparities persist even in settings where state-level insurance mandates guarantee coverage for fertility treatment [14]. A growing body of data suggests that these trends also persist in fertility preservation. And, unfortunately, disparities in access to fertility preservation care extend to other patient characteristics including socioeconomic status, age, and parity [6,7,15,16,17]. This narrative review aims to provide a brief summary of the data and unanswered questions regarding disparities in fertility preservation among patients with ovaries. It highlights potential approaches to bridge the gap in the provision of fertility preservation care and future directions for further research.

## 2. Overview of Disparities Data

### 2.1. Racial and Ethnic Disparities

Disparities have been documented at all stages of the fertility preservation continuum of care, from referral and counseling to treatment. The odds of fertility preservation referral are approximately two times higher for white women [17]. Even in studies that did not find a significant association between race and referral to a reproductive specialist, racial minorities are underrepresented relative to the racial mix of the catchment area [18]. Among young people diagnosed with cancer between the ages of 15 and 35 years old, 40% of non-white respondents reported an unmet need for infertility information, compared to 28% of white respondents [19]. In a study of African American women with breast cancer, 45.8% reported being aware of the potential impact of cancer treatment on fertility and 56.3% reported that their providers discussed fertility with them [20]. Although not statistically significant, a fertility preservation discussion is noted to be twice as likely to be documented in the electronic medical record for white patients compared to patients of color [21,22]. Even among patients counseled regarding fertility preservation, Black, Native American, and Hispanic patients are significantly less likely to undergo treatment based on work carried out at multiple institutions across the country [6,16,23,24,25,26].

A survey of women under the age of 40 with a new diagnosis of breast cancer found that non-white race was significantly associated with increased concern about fertility, suggesting that these disparities are not due to lack of interest [27]. Interestingly, patients in racial and ethnic minority groups are more likely to undergo fertility-sparing treatment for gynecologic cancers. However, in the same dataset, use of assisted reproductive technology was low and was associated with non-Hispanic white race [28]. 

### 2.2. Socioeconomic Disparities

Socioeconomic disparities within reproductive care are vast, given the substantial financial burden of fertility treatments, which is exacerbated in the context of a new oncologic diagnosis. One large survey study at an academic medical center found that women with an annual income of <USD 30,000 were less likely to report receiving fertility preservation counseling; however, income was not associated with who ultimately underwent fertility preservation procedures [6]. However, other studies have shown that income did not predict whether a patient received fertility preservation counseling [18,29]. In a chart review of 806 reproductive-aged women at an academic medical center, average income as determined by home zip code did not differ between patients who underwent fertility preservation consultation and those who did not [17]. Additionally, the same study showed that average distance between the hospital and home address did not differ among those who underwent fertility preservation consultation and those who did not [6]. 

Patients from low socioeconomic groups may be uninsured or have public insurance, which limits their access to care in general and certainly to fertility preservation. In a large study of nearly 3000 adolescents and young adults with a cancer diagnosis at an academic medical center, those with public insurance or uninsured were significantly less likely to be referred for fertility preservation counseling by 7.1% and 4% points, respectively, when compared with patients with private insurance. However, insurance status is not necessarily predictive of who ultimately underwent a fertility preservation procedure [30]. While insurers rarely cover fertility preservation procedures, the initial consultation is often covered. Therefore, insurance status is not associated with likelihood of utilizing fertility preservation consultation [16]. However, one study demonstrated the opposite, with significantly more patients with private insurance receiving fertility preservation consultation (80.5% versus 61.4%) [17]. The data on whether patients who are publically insured, uninsured, or from lower socioeconomic groups have difficultly accessing fertility preservation is mixed. However, considering the high financial and time burden of treatment, it is easy to image that the barriers to fertility preservation are substantial. 

### 2.3. Age Disparities

A patient’s age may also impact their likelihood of being referred for or undergoing fertility preservation. One survey study found a trend towards patients older than 35 years old being less likely to pursue fertility preservation [6]. A retrospective study, showed that of the patients eligible for fertility preservation, patients less than 35 years were significantly more likely to undergo some type of fertility preservation [17]. While these trends are not unexpected, it is crucial to note that older patients within the reproductive age window arguably need fertility preservation counseling the most, as they are likely to be at the highest risk of losing their fertility potential after gonadotoxic therapy. 

The decreased fertility preservation utilization for older patients is likely multifactorial. Referring providers might assume that older women are less inclined to pursue fertility preservation due to their age. Alternatively, providers may avoid raising false hope, considering the well-known challenges associated with achieving positive outcomes with fertility preservation in older age groups. A retrospective review of 137 women who utilized autologous vitrified oocytes, cryopreserved for elective or non-oncologic medical indications, revealed that age played a significant role in fertility preservation success. Women aged 36 years or older exhibited lower clinical birth rates upon returning to use their vitrified oocytes than younger women [31]. This decline in success can be attributed to older women having fewer available oocytes for vitrification, as well as diminished reproductive competence with an increased aneuploidy rate [32]. According to a patient decision tool routinely used in counseling individuals seeking fertility preservation, a 34-year-old woman would need to freeze 10 oocytes to have a 75% chance of achieving at least 1 live birth, while a 37 or 42-year-old would need to freeze 20 or 61 oocytes, respectively, to have the same 75% chance [33]. Although success rates are undoubtedly lower in older women, individualized discussions are crucial to ensure that each patient can make an informed decision aligned with individual reproductive goals.

### 2.4. Parity Disparities

A patient’s family size at time of cancer diagnosis may influence their likelihood of being referred for fertility preservation counseling. It may not be surprising to find that women without children are more likely to undergo fertility preservation consultation [17]. However, in a cross-sectional study of 249 oncologists, 10% do not discuss fertility with their patients who already have children [7]. In a qualitative study that assessed oncologists’ practice patterns regarding fertility preservation consultation or referrals, several physicians stated that the number of children the patient currently has factors into their decision on whether to discuss fertility preservation [15]. However, not all studies highlight this disparity, and some studies do not find an association between having children and the likelihood of undergoing fertility preservation [16]. Patients with one child or more may desire more children to complete their family and the values surrounding their ideal family size should be assessed, not assumed, when faced with gonadotoxic therapy. 

## 3. Approaches to Bridge the Gap

### 3.1. Insurance Mandates

In discussions about access to fertility care, insurance mandates are often mentioned as a way to increase affordability and utilization of fertility treatment. While insurance mandates increase access to fertility treatment overall, they do not necessarily ameliorate racial disparities in treatment [14]. Given that insurance mandates to cover fertility care apply to private insurance plans, one possible explanation for persistent disparities may be insurance type, as 29.1% of Black patients and 21.8% of Hispanic or Latino patients have public health insurance, compared with 16.2% of white patients [34]. However, even among more ethnically diverse cohorts of patients with similar rates of private insurace coverage, white patients are far more likely to pursue fertility preservation than patients of color, indicating that insurance status alone cannot fully explain the discrepancy [35]. Therefore, legislative intervention in the form of insurance mandates should not be overlooked as an approach to the problem, but should perhaps be considered a basic component of what must clearly be a multifaceted strategy.

### 3.2. Decision Support

The literature identifies several factors that impede decision making about fertility preservation, including inadequate provision of fertility information, fear related to perceived risks of pursuing fertility preservation, non-referral, competing priorities, personal situation (e.g., parity, relationship status), and financial factors [36]. However, most studies exploring these issues lack the sample size necessary to uncover themes specific to the cultural needs and concerns of different racial groups or socioeconomic classes. While significant effort has been put into evaluating the thoughts and attitudes of oncologists, reproductive specialists, and patients regarding fertility preservation, these studies often do not stratify responses based on patient self-reported race [37]. Prior to investing in programs to increase fertility preservation participation among minority groups, it is essential to conduct research that specifically examines the perspectives of these patients. Employing community-based participatory research principles may be the most effective approach to building or restoring trust between healthcare institutions and the surrounding community, thereby increasing the likelihood of successful therapeutic relationships. Once community-specific factors that influence decisions regarding fertility preservation are identified, decision aids can be developed as tools to reduce decisional regret and enhance patient satisfaction with the information received [38,39].

### 3.3. Access to Information

It may be a viable option to address gaps in counseling by improving patient access to fertility preservation information. While information regarding fertility preservation is often available on the Internet, not all patients have Internet access, and much of the available information is written above the recommended reading level of the American Medical Association and the National Institutes of Health (6th–7th grade) [40,41,42]. Furthermore, language barriers are an issue; a review of fertility preservation information on the website of pediatric cancer programs found that 93.8% did not provide information in Spanish [43]. While providing accessible information is an important step, similar to insurance mandates, this strategy alone is unlikely to fully address disparities in fertility preservation care. 

### 3.4. Impact of Bias

Racial disparities in clinical settings are influenced by implicit bias that exists among healthcare professionals. This bias leads to inequities in healthcare and must be acknowledged [44,45]. One potential approach to addressing this issue is the implementation of an “opt out” referral system for fertility preservation, which would eliminate some of the impact of provider bias on which patients are seen for consultation and increase referrals overall. One study found that this strategy does indeed increase referrals, with patients 3.6 times more likely to be seen following implementation. However, in this study, even with this approach, there were still disparities in which patients ultimately underwent a preservation procedure, with none of the four Black patients or three Hispanic patients, and only one of four Asian patients who received consultation actually undergoing a procedure [46]. This suggests that further work is needed to address the impact of bias on patient decision-making after the consultation. Additionally, it is important to consider whether the current reproductive endocrinology workforce has the capacity to sustain an “opt out” system, given the time-sensitive nature of oncofertility referrals and long wait times for general infertility services in some health systems. To avoid introducing further inequities, additional resources in reproductive endocrinology may be necessary before implementing such a system.

### 3.5. Provider Education

One potential area of intervention to improve fertility preservation access is to improve healthcare provider education on available options, as the field is rapidly evolving. However, even with knowledge of the options, healthcare providers may still face discomfort in discussing fertility preservation with their patients, leading to further barriers to care. This discomfort may stem from uncertainty about available options, leading to a reluctance to refer to fertility specialists. Additionally, the treating oncologist may be guarded in their prognosis for patient survivorship and less likely to refer in these situations to avoid giving false hope. 

These barriers in provider education are echoed in the literature; a study analyzing qualitative data from interviews of adult and pediatric oncologists found that providers reported a lack of knowledge of fertility preservation due to having received no formal training in the subject. They also noted that discussions about fertility preservation added an extra layer of stress to an already burdensome situation for the patient [47]. One cross-sectional study of fertility preservation referral patterns of oncologists found that despite 90% of oncologists affirming that they were ‘very knowledgeable ‘or ‘aware of’ fertility preservation options, only 17% have experience with embryo cryopreservation, and 22% report experience with GnRHa [7]. Of those surveyed, 75% reported interest in attending an education seminar on currently available fertility preservation techniques. A qualitative study surveyed practices of 16 oncologists regarding how they handle fertility preservation discussions with their oncology patients. While most of the physicians surveyed stated they routinely discuss fertility preservation with their patients of childbearing age, interestingly, the five physicians with the highest volume reported that they were unware of fertility preservation techniques and the appropriate referral provider [15]. Similarly the most senior providers stated that do not typically discuss fertility preservation and are unfamiliar with how to refer patients [15]. 

Provider education is a significant gap in fertility preservation care and is not necessarily a topic that is formally covered in oncology fellowship [15]. Therefore, increasing healthcare provider education on fertility preservation options and techniques may help overcome some of the barriers to care and improve access to all patients. 

Another factor contributing to the limited oncologist education may be the lack of easily accessible IVF centers in their vicinity. According to the 2020 ART Fertility Clinic and National Summary Report, 499 clinics reported outcomes to the CDC [48]. While some of these clinics may be situated within academic settings with convenient connections to referring oncologists, the majority are likely private clinics. It is crucial for all IVF clinics, regardless of practice model and affiliation, to seize the opportunity to collaborate with oncology colleagues in their community, fostering increased education and referral opportunities for these vital fertility preservation services. 

### 3.6. Low-Cost and Expedited Consultation Efforts

Patients with a new cancer diagnosis are overwhelmed with information, and programs with opt-out or included consultations with fertility specialists can help give patients the information they need in order to make an informed decision regarding whether or not to proceed with consultation. However, as mentioned previously, although increasing referrals for fertility preservation for all reproductive-aged cancer patients may help alleviate disparities, it is important to consider the potential impact on the capacity of fertility specialists programs. Additionally, not all patients referred will be able to afford the fees associated with the consultation, let alone the treatment. One possible solution is for fertility specialists to utilize trainees and mid-level providers and offer free or discounted consultationss to provide initial patient education. 

We found that offering a no-charge telephone consultation conducted by a reproductive endocrinology fellow or nurse practitioner prior to a formal in-person consultation led to an increase in completed consultations for fertility preservation [49]. This allowed patients to make an informed decision before proceeding with formal consultation or testing that may result in significant out-of-pocket costs. While this low-cost solution may not be applicable to all centers without access to trainees or mid-level providers, it demonstrates that creative solutions to increase access and decrease cost burden to the patient can make a significant difference. 

Once a patient elects to proceed with fertility preservation, the associated costs can be a substantial burden, often not covered by insurance, and can preclude a patient from proceeding. A 2010 study assessed the average cost of fertility preservation at 154 reproductive clinic locations from consultation to procedure, finding that the average cost for egg or embryo cryopreservation is USD 8655. While cancer patients typically qualify for supportive programs or grants, such as Livestrong or Heartbeat, the resulting subsidized cost is still significant, especially considering the other financial and social stressors of a new oncologic diagnosis. To increase access to fertility preservation, increased support in assisting patients to apply for financial assistance programs and creative solutions to decrease costs should be considered. 

## 4. Conclusions

The circumstances that impact the decision of minority patient groups to pursue treatment represent the next frontier in fertility preservation research. As our exploration of the literature here has demonstrated, there are extensive gaps in our understanding of underrepresented minorities’ utilization of fertility preservation and why patients of a certain race, socioeconomic group, age, or family size are less often referred for care. In addition to the questions raised in this review, there is a relative paucity of data in the literature as it pertains to similar disparities in sperm-producing individuals. 

Given the missing pieces in our understanding, it is essential to directly engage with these groups to address disparities and promote equity. We need to ensure that those who have been historically excluded from the table are invited and encouraged to participate. It is time to ask ourselves if the table can be set in a way that is inclusive and welcoming for all.

**Figure 1:**
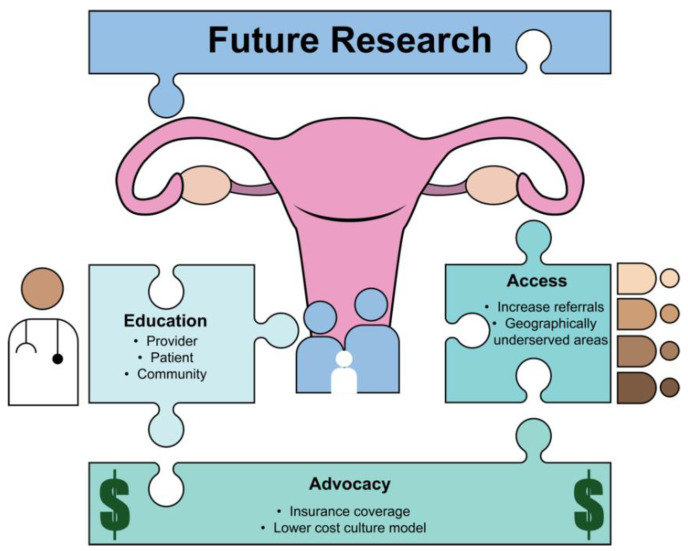
Recommendations to improve health disparities in fertility preservation.

Moving forward, it is clear that more research is needed to identify the factors that impact the decision-making process of underrepresented groups when considering fertility preservation. This research should focus on understanding the unique needs and experiences of these communities, and on developing interventions to address disparities in access to care. This will likely require a comprehensive approach that includes education and outreach, research, and policy changes (Figure 1). By working together to identify and address these disparities, we can ensure that all individuals access the care they need to achieve their reproductive goals after their cancer treatment. 

## Data Availability

Not applicable.
